# Type-I interferon pathway and DNA damage accumulation in peripheral blood of patients with psoriatic arthritis

**DOI:** 10.3389/fimmu.2023.1274060

**Published:** 2023-12-06

**Authors:** George E. Fragoulis, Panagiotis A. Ntouros, Adrianos Nezos, Nikolaos I. Vlachogiannis, Iain B. McInnes, Maria G. Tektonidou, Charalampos Skarlis, Vassilis L. Souliotis, Clio P. Mavragani, Petros P. Sfikakis

**Affiliations:** ^1^ Joint Academic Rheumatology Program, First Department of Propaedeutic and Internal Medicine, National and Kapodistrian University of Athens Medical School, Athens, Greece; ^2^ Institute of Infection, Immunity and Inflammation, University of Glasgow, Glasgow, United Kingdom; ^3^ Department of Physiology, National and Kapodistrian University of Athens Medical School, Athens, Greece; ^4^ Institute of Chemical Biology, National Hellenic Research Foundation, Athens, Greece

**Keywords:** psoriatic arthritis, type-I Interferon, DNA damage, PBMC (peripheral blood mononuclear cells), IL-6, IL-1

## Abstract

**Objectives:**

The abnormal DNA damage response is associated with upregulation of the type-1 interferon (IFN-I) pathway in certain rheumatic diseases. We investigated whether such aberrant mechanisms operate in psoriatic arthritis (PsA).

**Methods:**

DNA damage levels were measured by alkaline comet assay in peripheral blood mononuclear cells from 52 PsA patients and age-sex-matched healthy individuals. RNA expression of *IFIT1*, *MX1* and *IFI44*, which are selectively induced by IFN-I, was quantitated by real-time polymerase chain reaction and their composite normalized expression resulted in IFN-I score calculation. RNA expression of *IL1β*, *IL6*, *TNF*, *IL17A* and *IL23A* was also assessed in PsA and control subgroups.

**Results:**

In PsA, DNA damage accumulation was increased by almost two-fold compared to healthy individuals (olive tail moment arbitrary units, mean ± SD; 9.42 ± 2.71 *vs* 4.88 ± 1.98, p<0.0001). DNA damage levels significantly correlated with serum C-Reactive-protein and *IL6* RNA expression in PBMCs. Despite increased DNA damage, the IFN-I score was strikingly lower in PsA patients compared to controls (-0.49 ± 6.99 *vs* 4.24 ± 4.26; p<0.0001). No correlation was found between IFN-I pathway downregulation and DNA damage. However, the IFN-I score in a PsA subgroup was lower in those patients with higher *IL1β* expression, as well as in those with higher *TNF*/*IL23A* PBMCs expression.

**Conclusion:**

DNA damage in PsA correlates with measures of inflammation but is not associated with the IFN-I pathway induction. The unexpected IFN-I downregulation, albeit reminiscent to findings in experimental models of spondyloarthritis, may be implicated in PsA pathogenesis and explained by operation of other cytokines.

## Highlights

- DNA damage accumulation is increased in blood mononuclear cells of PsA patients
- Levels of DNA damage correlate with CRP levels in PsA
- Type-I Interferon pathway is paradoxically downregulated in PsA

## Introduction

Psoriatic arthritis (PsA) is a common inflammatory arthropathy, affecting about 25% of patients with psoriasis. It is heterogenous with many clinical manifestations, including arthritis, skin psoriasis, enthesitis, nail involvement and dactylitis. Comorbidities, such as obesity, cardiovascular and metabolic diseases, as well as mental health disorders, are commonly present (1). Although significant advances have been made in understanding underlying pathogenetic mechanisms, several issues remain ill defined. Moreover, different pathogenetic pathways could in part explain the observed diversity in clinical expression (2).

DNA damage has been shown by our and other groups to be increased in systemic rheumatic diseases (SRD) such as systemic sclerosis, systemic lupus erythematosus (SLE) and rheumatoid arthritis (RA) (3–6), possibly related to deficiencies in DNA repair machinery (4, 5). Increased DNA damage, in turn, has been linked to augmented type-I Interferon (IFN) responses (6, 7). Besides, IFN-I may play a role in the pathogenesis of psoriatic disease (8, 9). Polymorphisms in genes encoding for proteins involved in IFN-I response have been found in PsA patients (9) while there is some evidence that IFN-I pathway is up-regulated in the skin of patients with psoriasis (10, 11) and in the synovium or in the synovial fluid of patients with PsA (12, 13). However, IFN-I expression in the peripheral blood cells of PsA patients remains to be elucidated.

Therefore, we aimed to investigate the presence of DNA damage and an IFN-I signature (which corresponds to the evidence of an upregulation of transcripts induced by IFN-I) (14) in PsA and to determine whether subgroups of PsA patient exhibited differential signatures.

## Patients and methods

### Study cohort characteristics

Consecutive patients, fulfilling the CASPAR criteria for PsA, attending our outpatient’s rheumatology clinic from April 2020 to January 2021 were enrolled in the study. Age- and sex- matched healthy controls (HC) were included in the study, with no past medical history of SRD. Exclusion criteria for both groups included active (on chemo- radio- therapy) malignancy and active or recent (last 2 weeks) infection (self-reported but also confirmed by a negative CRP in healthy controls) or vaccination. The study was approved by the “Laiko” Hospital Ethical Committee (No 314.21) and all participants provided written informed consent.

### Cell isolation

Peripheral Blood Mononuclear Cells (PBMCs) were isolated immediately after blood sample collection using Ficoll gradient centrifugation as previously described (15). Cells were resuspended in Freezing Medium [90% Fetal Bovine Serum (FBS), 10% Dimethyl sulfoxide (DMSO)] or lysed in TRITidy G (AppliChem, Germany) and stored at -80°C until further processing.

### DNA damage measurement

Endogenous DNA damage levels in PBMCs were measured by single-cell gel electrophoresis (comet assay) under alkaline conditions, measuring single-strand breaks (SSBs) and/or double-strand breaks (DSBs) as previously described (15). Briefly, PBMCs (5x10^4^ cells) on comet slides were lysed and electrophoresis was performed for 30min at 1V/cm, 4°C. Slides were stained with SYBR Gold Nucleic Acid Gels Stain (Thermo Fischer Scientific, #S11494) and visualized using fluorescence microscope (Zeiss Axiophot). Comet images were analyzed by the Open Comet in ImageJ software. Olive tail moment (OTM) of at least 200 cells/treatment condition was evaluated in order to quantify DNA damage.

### RNA extraction, reverse transcription, and type-I IFN score quantification

Total RNA was extracted from PBMCs, using the TRITidy G Reagent (AppliChem, Germany) as per the manufacturer’s instructions, and immediately stored at -80°C. The quantity and quality of RNA samples were spectrophotometrically tested (Biospec Nano, Japan).

One microgram of RNA was reverse transcribed into complementary DNA (cDNA) with Superscript III (Thermo Fisher Scientific, USA). cDNA samples were diluted 1:10 with nuclease-free water (AppliChem, Germany) immediately after synthesis and stored at -20°C.

Quantitative real-time polymerase chain reaction (qRT-PCR) was used to quantify the expression of selected genes using the Bio-Rad IQ5 thermocycler and the KAPA SYBR FAST Mastermix (KAPABiosystems, South Africa). Genes preferentially induced by type-I IFNs were selected and included the following: IFN-induced protein with tetratricopeptide repeats 1 (IFIT1), myxovirus (influenza virus) resistance 1 (MX1) and Interferon Induced Protein 44 (IFI44). As an internal control and normalization gene (housekeeping gene), we used the glyceraldehyde phosphate dehydrogenase (GAPDH).

To assess the IFN-I signature, we calculated the PBMC type-I Interferon score (IFN-I score) as a composite of three type-I Interferon-inducible genes (IFIT1, MX1 and IFI44) normalized to the house-keeping gene (GAPDH), as previously described (6, 15, 16). In detail, a reference sample was included in each PCR plate to ensure normalization across experiments. Briefly, the qRT-PCR was performed in a total volume of 25μL/reaction (2 μL of template cDNA, 0.4 μM of each primer, 12.5μl 2× IQ SYBR Green SuperMix (Bio-Rad), sterile water). The amplification protocol started with 95°C for 4 min followed by 40 cycles at 95°C for 10 s and 60°C for 30s and 72°C for 30s. The product specificity was assessed by the melting curve analysis. The threshold value of each sample was obtained in the logarithmic portion of the amplification curve. All reactions were performed in duplicate. Each sample’s threshold values for the type-I IFNs and house-keeping genes were subtracted from the corresponding reference value and then divided by the house-keeping gene values of each sample, resulting to the relative expression value of each examined sample. Type I IFN score was defined as the sum of the relative expression of the three type-I Interferon-inducible genes (IFIT1, MX1 and IFI44). In a subgroup of patients (for which amount of RNA was available for further analysis, n=34), the expression of the genes encoding for TNF, Interleukin (IL)-1, IL-6, IL-23 and IL-17 was also examined via qRT-PCR (primers are presented in **Supplementary Table 1**).

### Statistical analysis

Normal distribution was examined by D’Agostino-Pearson and Shapiro-Wilk tests. Continuous variables are presented as mean ± SD. Comparisons were performed with the use of Mann-Whiney U test and unpaired t-test for not normally and normally distributed parameters, respectively. Spearman’s test was used to examine correlations. If both parameters were normally distributed, Pearson test was used, instead. The level of statistical significance was set at p<0.05. Statistical analysis was performed in SigmaPlot, SPSS v.26 (IBM, USA) and GraphPad Prism 5.00 (GraphPad Software, Inc., USA).

## Results

### Cohort description

Fifty-two PsA patients were included in our study. Of subjects with PsA, 61.5% were female, 42.3% were current smokers. Their mean ± SD age, Body Mass Index (BMI) and disease duration was 52.8 ± 10.7 years, 28.9 ± 7.2 Kg/m^2^ and 82.9 ± 107.5 months, respectively. Further characteristics of patients included in the study are depicted in **Supplementary Table 2**.

### Increased endogenous DNA damage in PsA patients

First, we assessed DNA damage by measuring DSBs and/or SSBs via alkaline comet assay. We observed that in PBMCs of PsA patients, endogenous DNA damage levels are increased by almost 2-fold compared to healthy controls (mean ± SD; 9.42 ± 2.71 Vs 4.88 ± 1.98, p<0.0001) (**Supplementary Figure 1**). Next, we examined possible clinical and serological associations with measured DNA damage in PsA patients (**Tables 1**, **2**). Endogenous DNA damage levels were found to significantly correlate only with CRP levels (r=0.354, p=0.012) suggesting a relationship to intercurrent systemic inflammation (**Table 2** and **Supplementary Figure 2**) It has to be noted however, that CRP was increased (>5mg/l) in only 20% of our patients (**Supplementary Table 2**).

**Table 1:** Associations of demographical, clinical and laboratory factors with endogenous DNA damage and Type-I interferon scores and in patients with Psoriatic Arthritis.

Disease Features (n=52)	DNA damage score			Type-I Interferon score		
Demographics	Presence	Absence	p-value	Presence	Absence	p-value
	mean ± SD			mean ± SD		
Female gender (n=32)	9.13 ± 2.47	9.88 ± 3.07	0.339	-0.30 ± 7.20	-0.82 ± 6.82	0.992
Smoking (n=22)	8.93 ± 2.63	9.78 ± 2.77	0.357	0.25 ± 8.10	-1.05 ± 6.15	0.831
Clinical features Ever present	mean ± SD		p-value	mean ± SD		p-value
Enthesitis (n=18)	8.89 ± 2.51	9.70 ± 2.81	0.306	-2.48 ± 4.41	0.55 ± 7.90	0.083
Dactylitis (n=15)	9.08 ± 2.82	9.56 ± 2.69	0.572	1.36 ± 9.25	-1.25 ± 6.83	0.592
Nail disease (n=33)	9.44 ± 2.78	9.38 ± 2.67	0.943	0.28 ± 8.33	-1.86 ± 3.49	0.902
Axial disease* (n=24)	9.54 ± 2.55	9.30 ± 2.91	0.748	-1.34 ± 4.91	0.34 ± 8.61	0.898
Uveitis (n=2)	8.59 ± 5.25	9.47 ± 2.57	0.190	14.00 ± 12.74	-1.39 ± 5.59	**0.02**
IBD (n=4)	11.77 ± 3.03	9.22 ± 2.63	0.191	-2.21 ± 2.78	-0.36 ± 7.23	0.987
Clinical features Current^¶^	mean ± SD		p-value	mean ± SD		p-value
Enthesitis (n=10)	9.51 ± 2.33	9.40 ± 2.82	0.908	-2.56 ± 4.89	-0.01 ± 7.37	0.147
Dactylitis (n=4)	8.97 ± 2.79	9.46 ± 2.73	0.732	1.43 ± 5.73	-0.66 ± 7.11	0.311
Nail disease (n=26)	9.34 ± 2.76	9.51 ± 2.71	0.942	0.03 ± 7.58	-1.07 ± 6.41	0.660
BSA=0 (n=33)	9.56 ± 2.71	9.17 ± 2.77	0.594	-2.44 ± 3.90	2.88 ± 9.62	**0.05**
MDA (n=25)	9.57 ± 2.97	9.28 ± 2.51	0.706	-0.16 ± 5.81	-0.81 ± 8.04	0.264
Current Treatment	mean ± SD			mean ± SD		p-value
Steroids (n=16)	9.86 ± 2.82	9.22 ± 2.68	0.440	-2.36 ± 6.05	0.33 ± 7.30	0.104
cDMARDs (n=26)	9.16 ± 2.65	9.68 ± 2.80	0.475	-0.91 ± 6.24	-0.08 ± 7.78	0.552
Apremilast (n=2)	11.14 ± 2.89	9.31 ± 2.70	0.217	-0.32 ± 7.14	-3.47 ± 2.87	0.455
TNFi (n=23)	9.12 ± 3.28	9.74 ± 1.95	0.634	-0.18 ± 6.26	-0.77 ± 7.67	0.388
IL-23/17i (n=6)	10.12 ± 2.49	9.32 ± 2.76	0.469	1.20 ± 10.65	-0.76 ± 6.38	0.793

Statistically significant values are noted with bold fonts.

BSA, body surface areal IBD, inflammatory bowel disease; MDA, minimal disease activity; cDMARDs, conventional disease modifying anti-rheumatic drugs; TNF, tumor necrosis factor.

* axial disease: when both of the following were present: inflammatory axial symptoms and radiological findings in x-ray or MRI of the sacroiliac joints or the spine. ^¶^At the time of the enrolment in the study.

**Table 2:** Correlations of demographical, clinical and laboratory factors with endogenous DNA damage and Type-I interferon scores and in patients with Psoriatic Arthritis.

Characteristics	DNA damage score		Type-I Interferon score	
	r	p-value	r	p-value
Age	0.225	0.156	0.057	0.689
BMI	0.053	0.710	-0.430	**0.001**
Disease duration	0.098	0.489	0.145	0.306
DAPSA	0.078	0.588	0.092	0.524
BSA	-0.100	0.479	0.268	**0.05**
ESR	-0.001	0.996	0.163	0.263
CRP	0.354	**0.012**	-0.090	0.534

Statistically significant values are noted with bold fonts.

BMI, body mass index; BSA, body surface area; DAPSA, disease activity in psoriatic arthritis; ESR, erythrocyte sedimentation rate; CRP, C-reactive protein.

### Type I IFN signature in PsA patients

**Figure 1:**
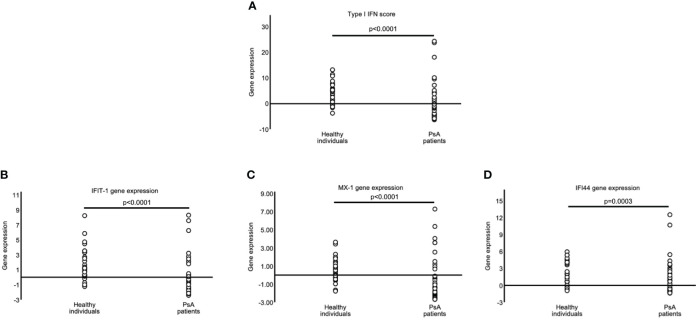
Decreased Type-I interferon scores in PsA patients. Graphs showing the type-I IFN score **(A)** in PBMCs of healthy controls (HC) (n=34) and Psoriatic Arthritis (PsA) patients (n=52) as a combination of the relative mRNA expression of 3 type I IFN–inducible genes (IFIT1, MX1, and IFI44) [**(B–D)**, respectively] with RT-qPCR. P-value is derived from Mann-Whitney U test.

Next, we investigated whether patients with PsA exhibit an altered type-I Interferon signature. Unexpectedly, we observed a strikingly lower IFN-I score in PsA PBMCs compared to the health control samples (mean ± SD; -0.49 ± 6.99 Vs 4.24 ± 4.26; p<0.0001) (**Figure 1A**). This differential type I interferon signature was evident also for each interferon inducible gene separately (IFIT1: mean ± SD; -0.49 ± 2.44 Vs 1.66 ± 2.10, p<0.0001, MX1: -0.80 ± 2.22 Vs 0.44 ± 1.29, p<0.001, IFI44: 0.81 ± 2.81 Vs 2.14 ± 2.05, p<0.0003) (**Figures 1B–D**).

### Lower IFN-I score in PsA patients: association with demographic, clinical and serological factors

We searched for possible associations between IFN-I score and demographic, clinical and serologic parameters. We found that the IFN-I score inversely correlated with BMI (**Table 1**). Also, IFN-I score was higher for patients who had ever manifested eye involvement and those who had current psoriasis compared to those who did not (Body Surface Area [BSA]=0). Along the same lines, IFN-I score correlated positively with BSA (**Table 1**). No other associations were found with any of the parameters (including treatment being received) examined. Furthermore, also unexpectedly, no association was detected between endogenous DNA damage and IFN-I score (r=-0.04, p=0.791). Conducting sensitivity analyses, we tested whether there was a correlation between DNA-damage and IFN-I score in subgroup of patients; No significant association was found for patients having active psoriatic lesions (p=1.000, r=0.001), for patients without active psoriatic lesions (p=0.798, r=-0.046), for patients who were bDMARD-naïve (p=0.672, r= -0.093), or for patients who had CRP values above 5mg/l (p=0.796, r=0.091).

### Associations between IFN-I score and other cytokines in PsA patients

Attempting to explain the dissociation between DNA damage and IFN-I score, we examined in a subset (n=34) of our cohort, for expression of key cytokines involved in the pathogenesis of PsA and for possible associations between their expression and IFN-I score. Demographic clinical and treatment characteristics as well as IFN-I scores did not differ between this subset of patients and the total cohort (n=52) (**Supplementary Table 3**). First, compared to healthy individuals, the RNA expression of *IL1* and *IL6* were found to be higher in PsA patients ([mean ± SD] 15.8 ± 23.0 vs 83.22 ± 219.0, p=0.01 and 3.50 ± 3.12 Vs 6.3 ± 15.9, p=0.02, respectively. This was not the case for *IL17* (1.25 ± 0.66 vs 0.98 ± 0.60, p=0.24), *IL23* (1.02 ± 0.43 vs 1.12 ± 0.84, p=0.55) and *TNF* (1.60 ± 0.50 vs 7.0 ± 20.60, p=0.08). (**Supplementary Figure 3**) Additionally, RNA expression of *IL23* were correlated with that of *TNF* (p<0.001, r=0.542) and RNA expression of *IL1* was correlated with that of *IL6* and *IL23* (p<0.038, r=0.357 and p<0.025, r=0.384, respectively) (**Supplementary Figure 4**). Of note, RNA expression of *IL6* but not of other cytokines tested, was correlated with DNA damage levels (p<0.017, r=0.406) (**Supplementary Figure 5**).

**Figure 2:**
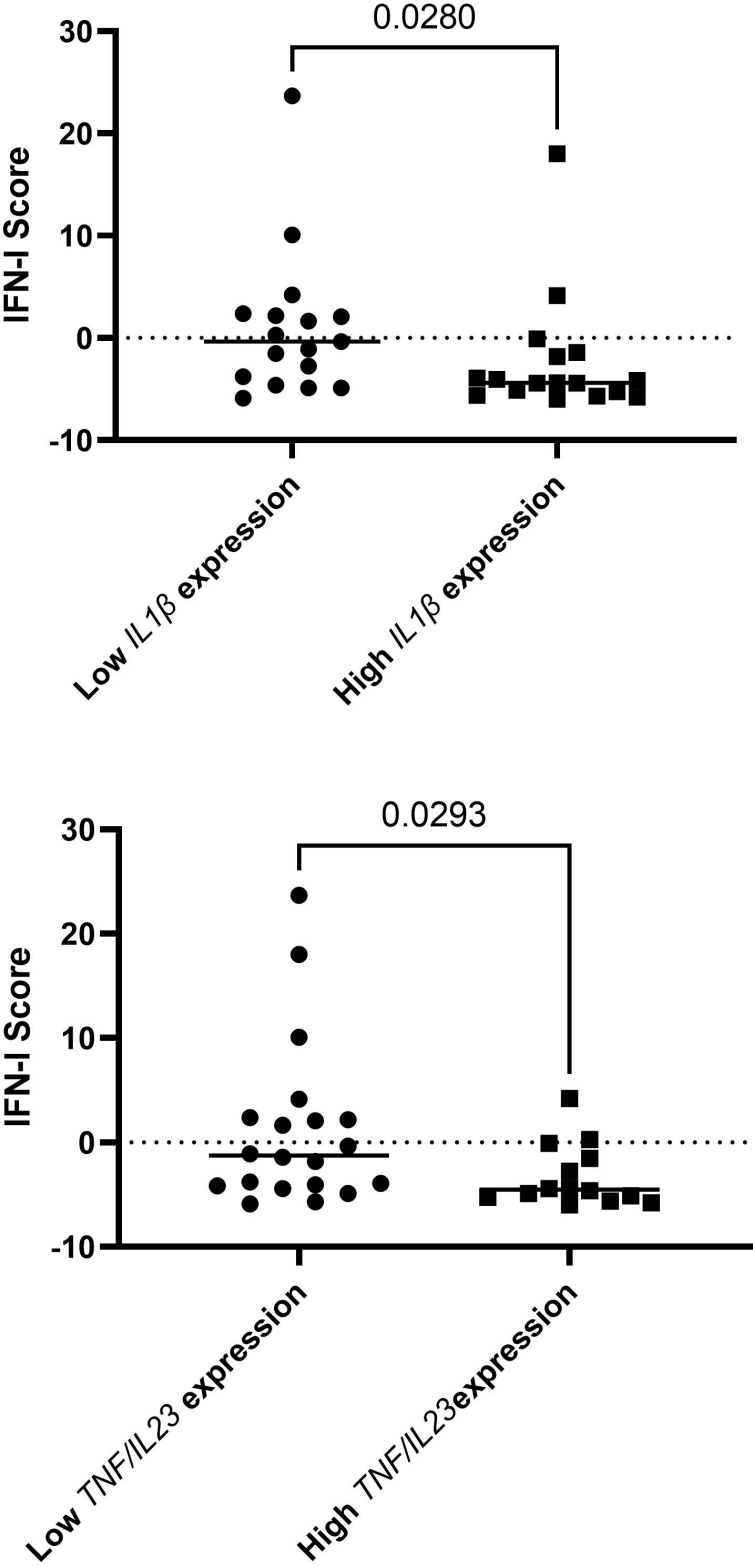
Decreased IFN-I scores in patients with higher *IL1*, compared to those with lower *IL1* RNA expression and in patients with both *TNF* and *IL23* high RNA expression, compared to the rest of the samples examined. In total, 34 patients were included in this analysis.

Assessing both IFN-I score and expression of cytokines as continuous variables, no statistically significant correlations were identified. However, when the expression of cytokines were divided to higher or lower (with cut-off being the median), we found decreased IFN-I scores in patients with higher *IL1*, compared to those with lower *IL1* RNA expression (-2.35 ± 5.85 vs 0.98 ± 7.11, p=0.028) and in patients with both *TNF* and *IL23* high RNA expression, compared to the rest of the samples examined, (-3.34 ± 2.89 Vs 1.42 ± 7.98, p=0.029) (**Figure 2**).

## Discussion

Pathogenetic mechanisms operating in PsA are ill defined thus far. We hypothesized that DNA damage, which has been found to be increased in other SRD, and subsequent inducible IFN-I expression could be aberrant in PsA. Using a well validated assay (5, 6, 15), we showed that there is increased DNA damage in PsA, which is more pronounced in patients with high CRP. This is also reflected in our finding that in our cohort, DNA-damage levels correlated with *IL6* expression. Besides, as it has been shown in oncology-studies, there is a relationship between *IL6* expression and DNA-damage. It seems that DNA damage can induce *IL6* expression via stimulator of interferon genes (STING) mediated NF-kB activation and also that IL-6 can affect DNA damage responses (17, 18). Interestingly, in PsA, only some patients display high CRP despite having active disease, possibly suggesting that additional pathogenetic mechanisms may operate. In our cohort, similar to other studies (19, 20) about 20% of the patients had increased (>5mg/l) CRP. The data described herein suggest that inflammation, in at least a subset of patients in whom CRP is elevated (20), may be associated with DNA damage – perhaps reflecting the burden of inflammatory disease and acquired or cumulative target tissue damage or stromal turnover.

Several data support the involvement of IFN-I in PsA. A recent study examining genetic profile in a large number of patients with PsA, found that polymorphism in PTPN22 was associated with PsA but not psoriasis (9). PTPN22 encodes for a protein tyrosine phosphatase inhibiting signaling in T cells and it has been found to mediate, via Toll-like receptors, IFN-I response, suppressing arthritis and colitis in mice models (21). Furthermore, although results are inconsistent (22), an IFN-I signature has been shown to be overexpressed in PsA synovium (12, 13). Dolcino et al, using Affymetrix arrays, have found in samples from 10 patients that expression of type I interferon inducible genes is increased in treatment-naïve patients, compared to healthy subjects, in paired synovium and peripheral blood of patients with PsA (13). Citrullinated and carbamylated peptides to LL-37 which is associated with increased IFNα production (23) are increased in PsA compared to osteoarthritis (12). In fact, in that study, IFNa was detectable by ELISA in 8/20 PsA synovial fluids tested, but in none of 12 synovial fluids obtained by individuals with OA. Also, staining with the protein MxA, which acted as a surrogate marker of the local IFN-I production, it was shown that this was abundant in PsA synovial tissue but not in that of OA (12). Finally, the IFN-I pathway seems to be activated in psoriatic skin, as attested by increased expression of molecules involved in this pathway, like Interferon regulatory factor (IRF)-7, IRF-9, MxA and (2–5) synthetase (24, 25). On the other hand, it seems that triggering with IFNa is not adequate to induce psoriasis phenotype neither in HaCaT keratinocytes nor in cultured skin biopsies (25). Furthermore, IFNa does not appear to be overexpressed in psoriatic skin. However, IFNa was found to be expressed from plasmacytoid dendritic cells (pDCs) in developing lesions of psoriasis (from skin obtained from marginal zones of spreading psoriasis lesions) (26).

Increased DNA damage has been shown to lead to increased IFN-I expression via the cGAS-STING (stimulator of interferon genes)-IRF3 pathway. More specifically, mice with deletion of the central DNA repair sensor Atm accumulated unrepaired DNA lesions, which induced a potent type I IFN response through the release of damaged DNA into the cytoplasm, where it activated the cGAS/STING pathway (6, 7). Another mechanistic link between increased DNA damage and induction of a cGAS/STING-mediated systemic immune responses is the generation of micronuclei, which make self-DNA accessible to the cGAS/STING sensors (27, 28). Interestingly, increased frequency of micronuclei has been reported in the cells of patients with various systemic autoimmune diseases (29). Finally, we have previously shown that increased DNA damage is associated with increased type I IFN-induced gene expression in PBMCs of patients with systemic sclerosis (6).

Given that increased DNA damage has been shown to lead to increased IFN-I expression, and also that IFN-I seems to be a player in pathogenesis of PsA (8, 9), we expected to find IFN-I signature to be augmented in our patients. To assess IFN-I signature, we calculated the type-I Interferon score (IFN-I score) as a composite of three type-I Interferon-inducible genes (IFIT1, MX1 and IFI44). Although, these genes can be upregulated also by other cytokines, including IFN-γ, they are mainly induced by type-I IFN (30–34).

In contrast to our expectations, it was found that IFN-I signature was several-fold lower compared to healthy individuals. Of note, the difference remained statistically significant (p=0.003) even after adjusting for BMI (which was found to be inversely correlated with IFN-I score in PsA patients). For the assessment of IFN-signature, we opted to use the combined expression of three IFN-I-inducible genes. These 3 genes have been previously identified as IFNα/β-inducible by *ex vivo* treatment of healthy subject-derived whole blood cells with 10 different IFN-α subtypes and IFN-β, while at the same time being overexpressed in peripheral blood cells of SLE patients (35), the prototypical systemic autoimmune disease characterized by elevated type I IFN responses. Moreover, treatment of human WISH epithelial cell line with either recombinant IFNα or plasma derived from SLE patients upregulated the expression of these 3 genes (36), suggesting a direct association between the calculated type I IFN score and IFN-α pathway activity. More importantly, the 3 genes have been previously used as part of the reported type I Interferon signature in patients with multiple systemic autoimmune diseases (6, 15, 16, 37), as well as to determine response in clinical trials of IFN-α blockade in SLE (35).

This method (6, 15, 16, 37), which in contrast to the measurement of IFN with ELISA is not directly affected by factors like anti-IFN antibodies or lack of detection of the various IFN species by the antibodies used in the ELISA (37).

The mechanistic reasons for the lack of association between the accumulation of DNA damage and a prominent type I IFN signature in PsA PBMCs, as observed in other SRDs such as SSc (6), are currently unexplained but point to dysregulation of normal innate immune regulation, which seems to be affected in PsA (38). Interestingly, in our cohort, high IL-1 expression was inversely associated with IFN-I expression, in agreement with the hypothesis of the negative cross-regulation between IFN-I and IL-1 expression (39, 40). There are other data also supporting the notion that IFN-I is suppressed in spondyloarthritis (SpA). In B27 transgenic mice, which serve as an SpA model, IFN-related genes were down-regulated (“reverse interferon signature”) (41). Interestingly, transcriptome analysis of B27 derived DCs showed a) an upregulation of suppressor of cytokine signaling-3 (SOCS3), known for its crucial role in limiting cytokine-mediated inflammatory responses, that may account for reverse IFN signaling and b) a down-regulation of IL-27, a cytokine that directly induces IFN production by various cell types, such as DCs, macrophages, NK cells, hepatocytes, and lung epithelial cells (42). Cantaert et al, examined 40 patients with spondyloarthritis (pathogenetic mechanisms of which are quite similar to that of PsA) and 20 healthy controls and found that the IFN-I signature was down regulated in the former. In this study, treatment with TNF inhibitors modulated IFN-1 activity (43), suggesting a counterbalance between TNF and IFN-I. In another study, examining patients with psoriasis, it was shown that treatment with adalimumab downregulated IFNa gene expression in the skin of the patients who responded to treatment at week 16 (44). Toward this direction, in our cohort patients who demonstrated high RNA expression of both *TNF* and *IL23A*, displayed low IFN-I scores. Taken all these together, one could support that pathways involving TNF, IL-23 and IL-1 might suppress the IFN-I in PsA.

Finally, in our cohort we found that IFN-I score was associated with psoriasis severity (assessed by body surface area) and inversely correlated with body mass index. Both observations are consistent with data from the existing literature suggesting that the IFN-I signature is robust in psoriatic skin (45) and that there is an impaired IFN-I response in people with higher BMI (46, 47).

We acknowledge our study has limitations. First, patients in our cohort have received various treatments including biologic DMARDs. No differences in DNA damage or IFN-I score were detected, however, between patients receiving different medications. It would be interesting to examine these parameters in treatment-naïve patients as our results cannot be extrapolated for these individuals. Along the same lines, CRP was elevated in only 20% of our patients, therefore the correlation of CRP with levels with DNA damage should be interpreted with caution. This, considering also the heterogeneity of PsA might be a plausible explanation for the differences between our findings and those reported by Dolcino et al. (13), in the study of which patients had not received treatment with DMARDs. Of note, in our study more patients (n=52 Vs n=10) were tested for IFN-I signature. Second, it has been suggested that IFN-I is increased in patients suffering from inflammatory arthritis, in earlier rather than later stages of the disease (48). In our study, disease duration did not seem to affect IFN-I expression. Third, ideally, we would like to test differential gene expression, simultaneously, at a tissue level. This has been assessed previously in 10 PsA patients (13), and it has been suggested that genes are modulated similarly at synovium and peripheral blood level. Additionally, we tested for the expression of cytokines having role in the pathogenesis of PsA, in a subgroup (n=34) of the total cohort (n=52). Of note, characteristics of patients did not differ between these two groups. Moreover, the number of individuals enrolled is not sufficient to draw firm conclusions and larger validation studies that would exploit a cytokine-based clustering of PsA patients would be of interest. A comprehensive study of DNA repair proteins and their potential association with IFN-I signature or proinflammatory gene expression would shed light in the involvement of the DDR pathway in immune aberrations in these patients, which unfortunately was not possible in our study due to limited biomaterial availability. Finally, further transcriptome-wide analyses like RNA-sequencing or microarray analysis to comprehensively study gene expression profiles in different PsA subpopulations can provide meaningful insights into disease pathogenesis, the effect of treatment on the transcriptomic profile of patients’ cells and the prognostic value of deregulated genes for disease progression or treatment response. Prospective RNA-seq. analysis of PsA patients’ blood transcriptome and its predictive value are a part of an ongoing project of our team. Of interest, a recent RNA-seq. study of PBMCs derived from PsA patients *vs* healthy controls showed a positive enrichment in PsA of several terms related to inflammation such as ‘inflammatory response’, ‘TNFa signaling via NFkB’, ‘complement’, ‘IL2 signaling’, and ‘IFNa and IFNg response’ (49) consistent with our results. Interestingly, longitudinal assessment of PBMC transcriptome revealed that transcriptomic alterations as soon as 1 month after treatment initiation could predict response at 6 months, with responders showing early downregulation of the proinflammatory gene signatures (49).

In conclusion, we show that DNA damage is increased in PsA patients, especially in the subgroup with higher CRP values which is also reflected in the correlation between the DNA damage and *IL6* expression. Unexpected IFN-I downregulation, albeit is reminiscent to findings in experimental models of spondyloarthritis, was observed regardless of CRP and treatment modalities and may be indicative of dysregulated innate immune regulation in PsA pathogenesis. It is possible that IFN-I expressing cells have been recruited to tissues leading to the reduced level of expression in blood. Whether IFN-I expression in the peripheral blood is suppressed by other proinflammatory molecules like TNF and IL-1 or is an intrinsic feature of patients with PsA warrants further studies.

## Data availability statement

The original contributions presented in the study are included in the article/**Supplementary Material**, further inquiries can be directed to the corresponding author/s.

## Ethics statement

The study was approved by the “Laiko” Hospital Ethical Committee (No 314.21) and all participants provided written informed consent. The studies were conducted in accordance with the local legislation and institutional requirements. The participants provided their written informed consent to participate in this study.

## Author contributions

GF: Conceptualization, Data curation, Formal analysis, Writing – original draft, Writing – review & editing. PN: Data curation, Formal analysis, Writing – original draft. AN: Formal analysis, Methodology, Writing – original draft. NV: Formal analysis, Methodology, Writing – review & editing. IM: Investigation, Writing – review & editing. MT: Formal analysis, Writing – review & editing. CS: Formal analysis, Methodology, Writing – original draft. VS: Investigation, Methodology, Supervision, Writing – review & editing. CM: Formal analysis, Supervision, Writing – review & editing, Conceptualization. PS: Formal analysis, Supervision, Writing – review & editing, Conceptualization.

## Supplementary material

Supplementary Figure 1
**(A)** Representative alkaline comet assay images of PBMCs from one healthy adult (A1) and one PsA patient (A2). **(B)** Dotplots representing the endogenous DNA damage levels (Olive tail moment arbitrary units) as assessed by alkaline comet assay in peripheral blood mononuclear cells (PBMCs) from healthy controls (HC) (n= 53) and PsA patients (n= 52). p-value is derived from Mann-Whitney U test. ^***^ P<0.001.

Supplementary Figure 2 Correlation of DNA damage levels (Olive tail moment arbitrary units) with CRP (mg/l) levels in the patients of our cohort (n=52), (p=0.012, r=0.354).

Supplementary Figure 3 RNA expression of *IL1β, IL6, TNFa, IL17A, IL23A* in peripheral blood mononuclear cells obtained from patients with psoriatic arthritis (n=34) and from healthy controls (n=9).

Supplementary Figure 4 Correlations between RNA expression of *IL1β, IL6, TNFa, IL17, IL23* in peripheral blood mononuclear cells obtained from patients with psoriatic arthritis (n=34). Axes are in log10 scale.

Supplementary Figure 5 Correlations between DNA damage, as assessed by olive tail moment (OTM) and RNA expression of *IL1β, IL6, TNFa, IL17, IL23* in peripheral blood mononuclear cells obtained from patients with psoriatic arthritis (n=34). Only correlation with *IL6* expression was statistically significant (p<0.017, r=0.406).
