# Supplementary figures and images for: Type-I interferon pathway and DNA damage accumulation in peripheral blood of patients with psoriatic arthritis
Source: Front Immunol. 2023 Dec 6;14:1274060. doi: 10.3389/fimmu.2023.1274060 (PMC10731026; doi:10.3389/fimmu.2023.1274060)

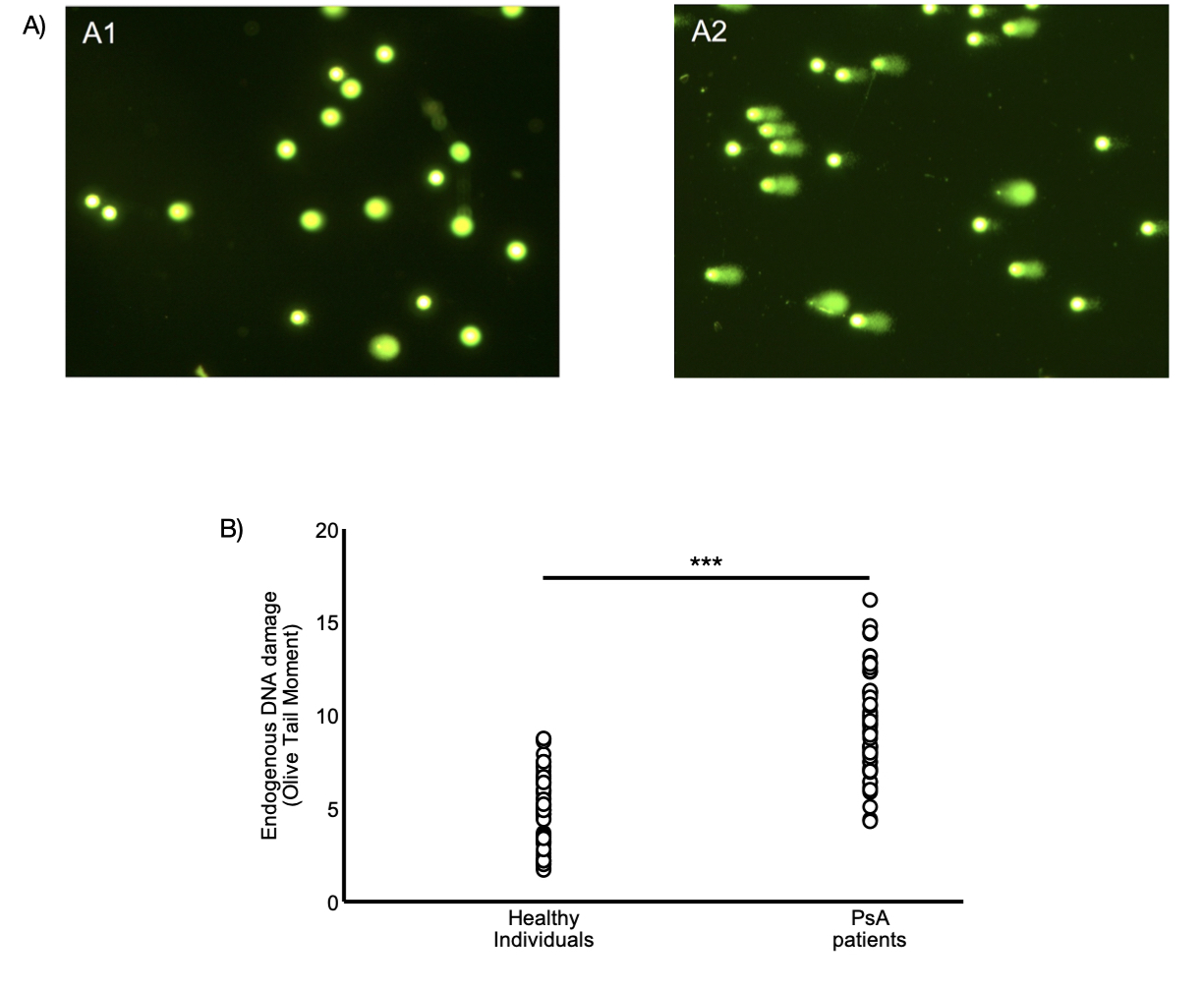

Supplement: Supplementary Figure 1 — (A) Representative alkaline comet assay images of PBMCs from one healthy adult (A1) and one PsA patient (A2). (B) Dotplots representing the endogenous DNA damage levels (Olive tail moment arbitrary units) as assessed by alkaline comet assay in peripheral blood mononuclear cells (PBMCs) from healthy controls (HC) (n= 53) and PsA patients (n= 52). p-value is derived from Mann-Whitney U test. *** P<0.001. [file Image_1.jpeg]

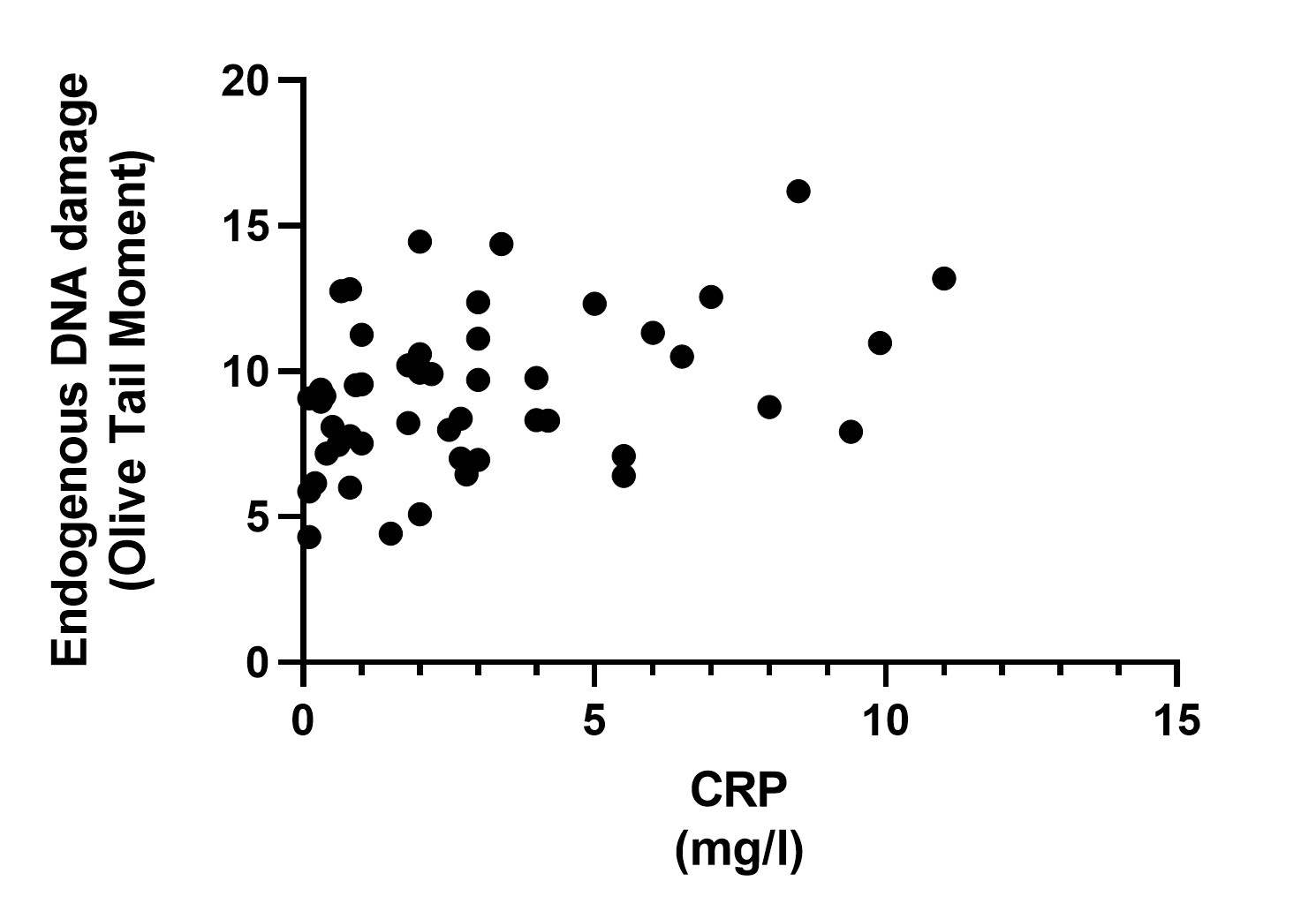

Supplement: Supplementary Figure 2 — Correlation of DNA damage levels (Olive tail moment arbitrary units) with CRP (mg/l) levels in the patients of our cohort (n=52), (p=0.012, r=0.354). [file Image_2.jpeg]

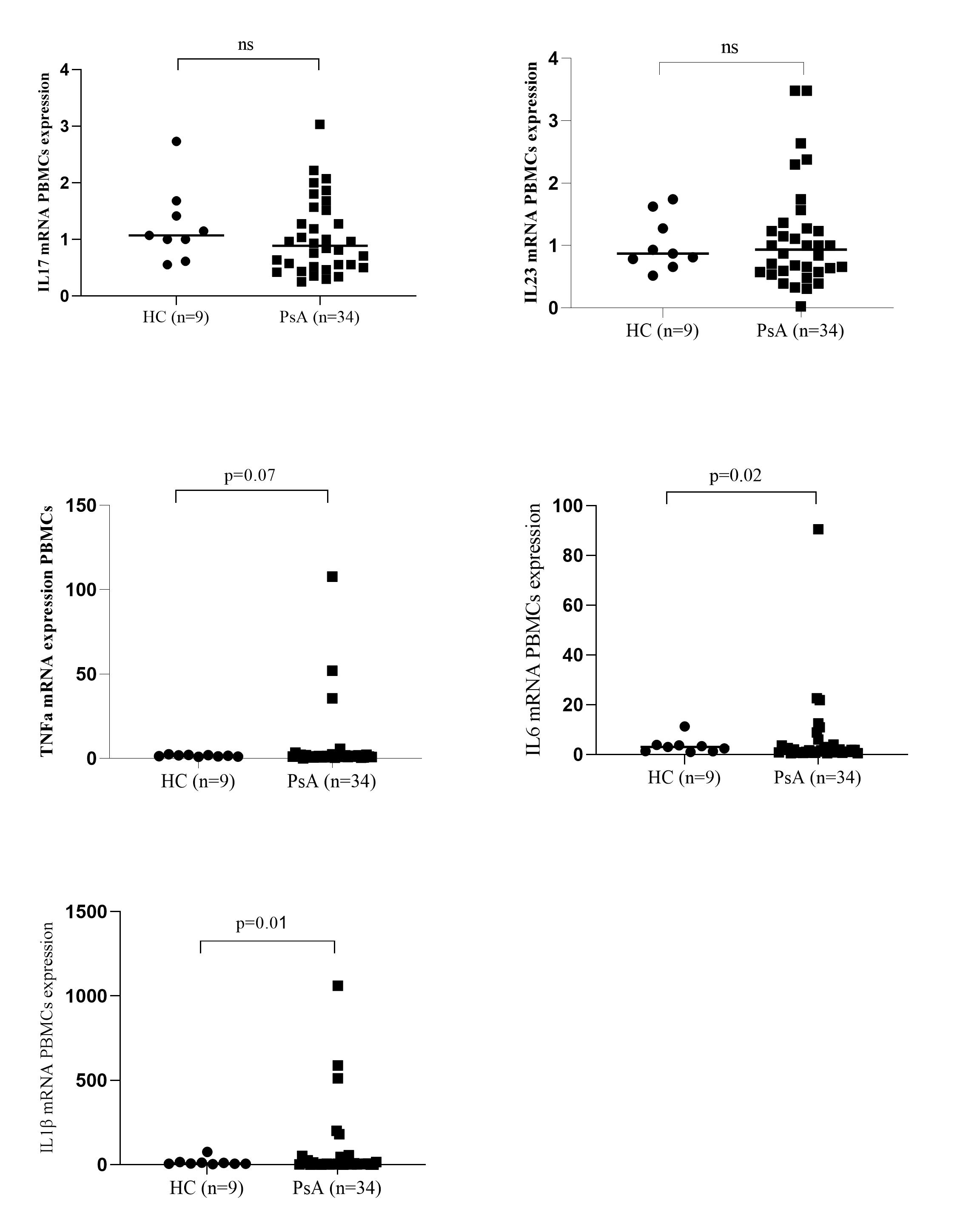

Supplement: Supplementary Figure 3 — RNA expression of IL1β, IL6, TNFa, IL17A, IL23A in peripheral blood mononuclear cells obtained from patients with psoriatic arthritis (n=34) and from healthy controls (n=9). [file Image_3.jpeg]

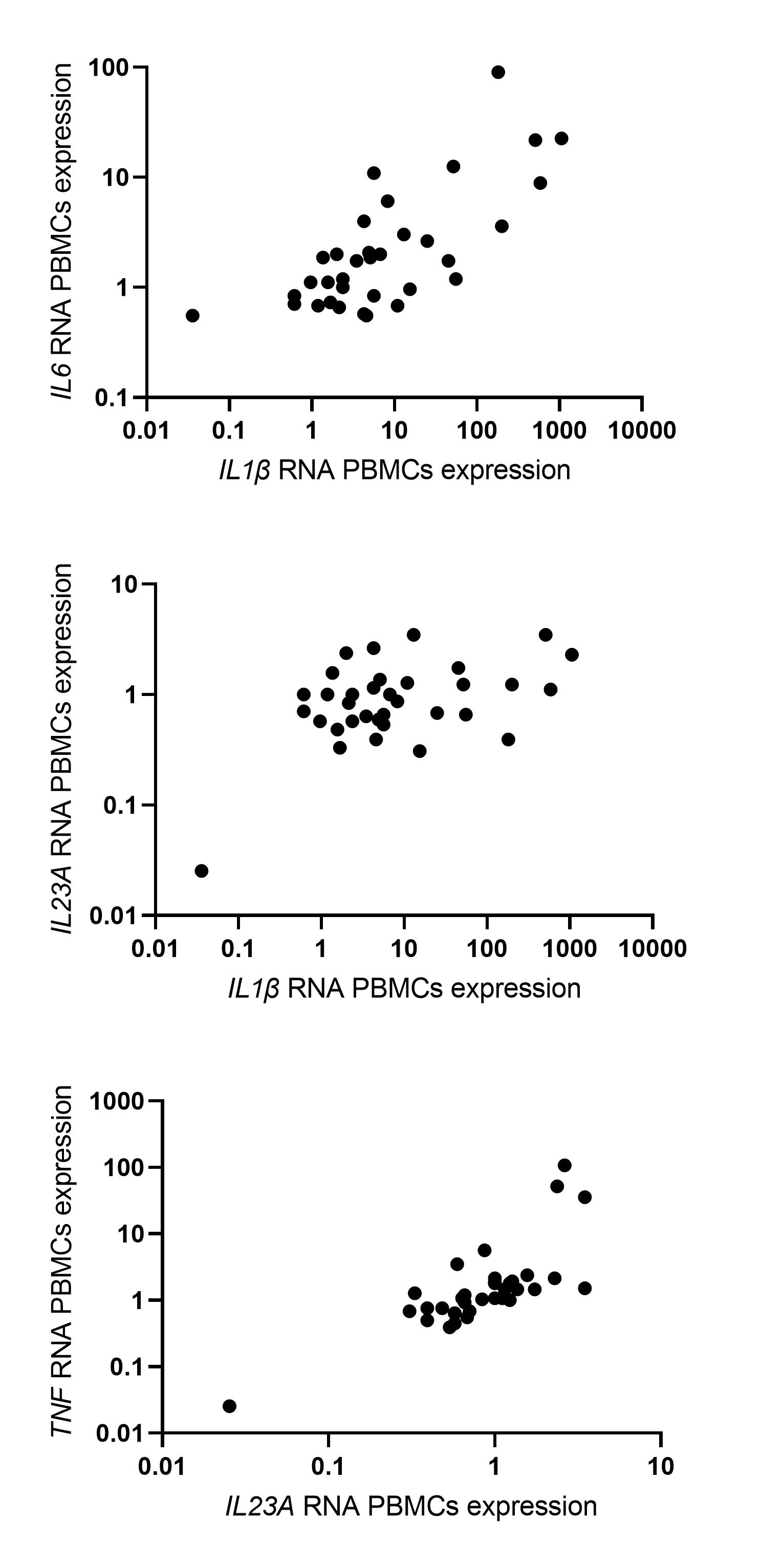

Supplement: Supplementary Figure 4 — Correlations between RNA expression of IL1β, IL6, TNFa, IL17, IL23 in peripheral blood mononuclear cells obtained from patients with psoriatic arthritis (n=34). Axes are in log10 scale. [file Image_4.jpeg]

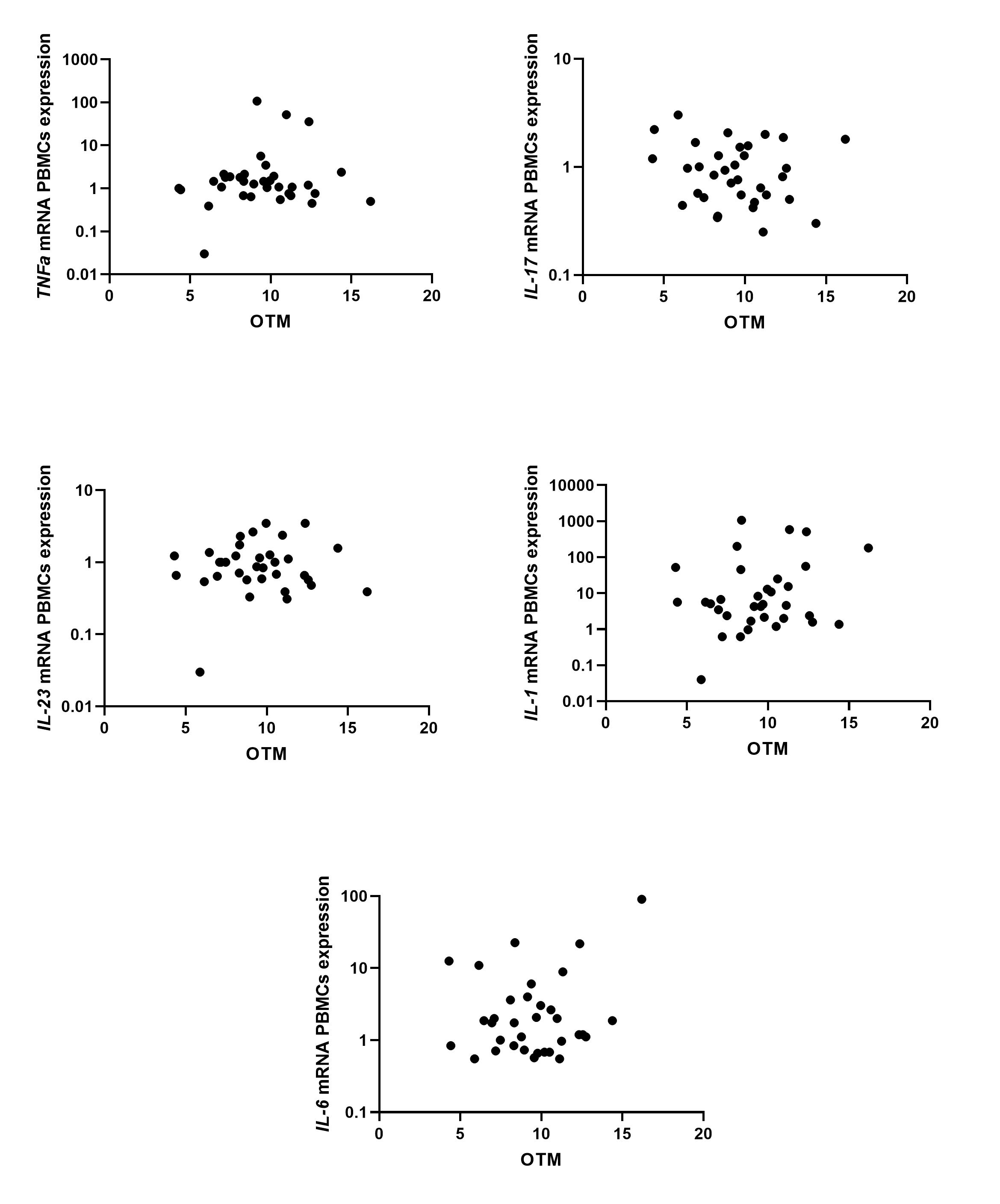

Supplement: Supplementary Figure 5 — Correlations between DNA damage, as assessed by olive tail moment (OTM) and RNA expression of IL1β, IL6, TNFa, IL17, IL23 in peripheral blood mononuclear cells obtained from patients with psoriatic arthritis (n=34). Only correlation with IL6 expression was statistically significant (p<0.017, r=0.406). [file Image_5.jpeg]
