# Supplementary material for: Type-I interferon pathway and DNA damage accumulation in peripheral blood of patients with psoriatic arthritis
Source: Front Immunol. 2023 Dec 6;14:1274060. doi: 10.3389/fimmu.2023.1274060 (PMC10731026; doi:10.3389/fimmu.2023.1274060)
Supplement: Supplementary file 6 [file Table_1.docx]

| **Gene** | **Forward primer sequence (5'🡪 3')** | **Reverse primer sequence (5'🡪 3')** |
| --- | --- | --- |
| GAPDH | CAACGGATTTGGTCGTATT | GATGGCAACAATATCCACTT |
| IFIT1 | CTCCTTGGGTTCGTCTATAAATTG | AGTCAGCAGCCAGTCTCAG |
| MX1 | TACCAGGACTACGAGATTG | TGCCAGGAAGGTCTATTAG |
| IFI44 | CTCGGTGGTTAGCAATTATTCCTC | AGCCCATAGCATTCGTCTCAG |
| IL23A | GAGCCTTCTCTGCTCCCTGATA | GACTGAGGCTTGGAATCTGCTG |
| IL6 | AGACAGCCACTCACCTCTTCAG | TTCTGCCAGTGCCTCTTTGCTG |
| IL17A | CGGACTGTGATGGTCAACCTGA | GCACTTTGCCTCCCAGATCACA |
| TNF | CTCTTCTGCCTGCTGCACTTTG | ATGGGCTACAGGCTTGTCACTC |

**Supplementary Table 1:** Specific genes and primers sequence for the gene expression analysis.

IFIT1: interferon-induced protein with tetratricopeptide repeats 1; IFI44: interferon-induced protein 44; MX-1: myxovirus (influenza virus) resistance 1; IL23:interleukin 23; IL6:interleukin 6; IL17: interleukin 17; TNFα: tumor necrosis factor-α; GAPDH: glyceraldehyde-3-phosphate dehydrogenase

| Demographic features | PsA  (n=52) | Healthy controls*  Group A  (n=53) | Healthy controls*  Group B  (n=34) |
| --- | --- | --- | --- |
| Age, mean ± SD (years) | 52.8 ± 10.7 | 52.8 ± 12.0 | 52.1 ± 8.9 |
| Female gender, n (%) | 32 (61.5) | 32 (60.4) | 20 (58.8) |
| BMI, mean ± SD | 28.9 ± 7.2 | 26.0 ± 2.5 | 27.4 ± 3.4 |
| Smoking (current), n (%) | 22 (42.3) | 19 (35.8) | 13 (38.2) |
| Follow-up time, mean ± SD (months) | 82.9 ± 107.5 | NA | NA |
| Clinical features (ever) | |  |  |
| Enthesitis, n (%) | 18 (34.6) | NA |  |
| Dactylitis, n (%) | 15 (28.8) | NA |  |
| Axial disease, n (%) | 24 (46.1) | NA |  |
| Nail disease, n (%) | 33 (63.5) | NA |  |
| DIP, n (%) | 2 (3.8) | NA |  |
| Eye involvement, n (%) | 2 (3.8) | NA |  |
| Bowel involvement, n (%) | 4 (7.7) | NA |  |
| Current laboratory features | |  |  |
| CRP >5mg/l, n (%) 10 (19.2) | |  |  |
| ESR>20mm/hr, n (%) 17 (32.7) | |  |  |
| Current treatment | |  |  |
| Steroids, n (%) | 16 (30.8) | NA |  |
| NSAIDs, n (%) | 9 (17.3) | NA |  |
| csDMARDs, n (%) | 26 (50) | NA |  |
| Apremilast, n (%) | 2 (3.8) | NA |  |
| TNF inhibitors, n (%) | 24 (46.2) | NA |  |
| IL-23 or IL-17 inhibitors, n (%) | 7 (13.5) | NA |  |

**Supplementary Table 2:** Characteristics of patients included in the study.

SD: standard deviation, n: number, DIP: distal interphalangeal joints, NSAIDS: non-steroidal anti-inflammatory drugs, NA: not applicable, cDMARDs: conventional synthetic disease modifying anti-rheumatic drugs, TNF: tumor necrosis factor, IL: Interleukin

* Group A: characteristics of healthy controls included in the experiment assessing the DNA-damage. Group B: characteristics of healthy controls included in the experiment assessing IFN-I expression.

| Demographic features | PsA  (n=34) | PsA  (n=52)  (total cohort) | Healthy controls  (n=9) |
| --- | --- | --- | --- |
| Age, mean ± SD (years) | 51.3 ± 10.4 | 52.8 ± 10.7 | 53.6 ± 10.2 |
| Female gender, n (%) | 19 (55.9) | 32 (61.5) | 5 (55.5) |
| BMI, mean ± SD | 28.5 ± 7.4 | 28.9 ± 7.2 | 26.3 ± 5.5 |
| Smoking (current), n (%) | 16 (47.0) | 22 (42.3) | 4 (44.4) |
| Follow-up time, mean ± SD (months) | 84.4 ± 107.5 | 82.9 ± 107.5 | NA |
| Clinical features (ever) | |  |  |
| Enthesitis, n (%) | 11 (32.4) | 18 (34.6) | NA |
| Dactylitis, n (%) | 10 (29.4) | 15 (28.8) | NA |
| Axial disease, n (%) | 19 (55.8) | 24 (46.1) | NA |
| Nail disease, n (%) | 23 (67.6) | 33 (63.5) | NA |
| DIP, n (%) | 2 (5.8) | 2 (3.8) | NA |
| Eye involvement, n (%) | 1 (2.9) | 2 (3.8) | NA |
| Bowel involvement, n (%) | 3 (8.8) | 4 (7.7) | NA |
| Current laboratory features | |  |  |
| CRP >5mg/l, n (%) 8 (23.5) | | 10 (19.2) | NA |
| ESR>20mm/hr, n (%) 10 (29.4) | | 17 (32.7) | NA |
| Current treatment | |  |  |
| Steroids, n (%) | 11 (32.4) | 16 (30.8) | NA |
| NSAIDs, n (%) | 7 (20.6) | 9 (17.3) | NA |
| csDMARDs, n (%) | 17 (50.0) | 26 (50) | NA |
| Apremilast, n (%) | 0 (0.0) | 2 (3.8) | NA |
| TNF inhibitors, n (%) | 16 (47.1) | 24 (46.2) | NA |
| IL-23 or IL-17 inhibitors, n (%) | 3 (8.8) | 7 (13.5) | NA |
| IFN-Score, mean ± SD | -0.68 ± 6.63 | -0.50 ± 6.70 | 0.35 ± 2.61 |

**Supplementary Table 3:** Characteristics of patients included in the subgroup where expression of cytokines was measured. Comparison with the total cohort (n=52) and with the healthy controls in which expression of cytokines was measured.

SD: standard deviation, n: number, NA: not applicable.
